# Supported Decision-Making and Paradigm Shifts: Word Play or Real Change?

**DOI:** 10.3389/fpsyt.2020.571005

**Published:** 2021-01-11

**Authors:** Jill Stavert

**Affiliations:** Centre for Mental Health and Capacity Law, School of Health and Social Care, Edinburgh Napier University, Edinburgh, United Kingdom

**Keywords:** mental health law, mental capacity law, CRPD, supported decision-making, Scotland, paradigm shift

## Abstract

Article 12(3) CRPD requires states parties to provide access by persons with disabilities to the support they may require in exercising their legal capacity. This is to ensure that the rights, will and preferences of persons with disabilities are enjoyed on an equal basis with others [Articles 12(1)(2) and (4) CRPD]. Moreover, the Committee on the Rights of Persons with Disabilities has made it clear that supported decision-making must replace substitute decision-making arrangements as these are discriminatory and deny equal enjoyment of the right to exercise of legal capacity for persons. At the same time, there is ongoing debate as to whether or not the absence of substitute decision-making regimes is essential for the non-discriminatory realization of an individual's rights, will and preferences to be achieved. To resolve this debate, however, specific attention needs to be paid to the CRPD message on what it actually means to give effect to the equal and non-discriminatory enjoyment of rights for all. In the context of persons with mental disabilities this requires looking beyond human rights simply in terms of limiting unwarranted interventions to the proactive removal of obstacles to full rights enjoyment and the creation of environments that respect and support such enjoyment. With this in mind this paper will therefore critically consider the use of supported decision-making within existing substitute decision-making regimes with particular reference to Scotland's mental health and capacity laws. It will consider the challenges this poses and whether it is indeed possible to adapt existing regimes to achieve CRPD compliance. In doing so, it is suggested that a full appreciation of the overarching CRPD message about equality and non-discrimination in the enjoyment of rights is required to bring about such compliance.

## Introduction

As is the case for many jurisdictions Scotland is wrestling with the issue of giving meaningful effect to the Convention on the Rights of Persons with Disabilities (CRPD), particularly its Article 12, and the extent to which this is possible. Indeed, whilst some of the conceptual and practical aspects of giving effect to Article 12 are unique to Scotland many other conceptual, and some practical, aspects are common to most states. A consideration of these issues are therefore of value to those seeking to bring about CRPD related change in Scotland and also in other jurisdictions.

Article 12(2) CRPD requires that “States Parties shall recognize that persons with disabilities enjoy legal capacity on an equal basis with others in all aspects of life.” In interpreting this right the Committee on the Right of Persons with Disabilities (CRPD Committee) General Comment No. 1 makes a distinction between “legal capacity” and “mental capacity” (1). Having legal capacity is the ability to, for example, make contracts, refuse medical treatment and marry. It is the ability to both be a rights holder and to exercise those rights and allows a person's views—their will and preferences—to be given effect and respected. Mental capacity, on the other hand, is a person's decision-making skills and which may vary from time to time and between individuals.

The CRPD Committee emphasizes that exercising legal capacity is something all human beings must enjoy on an equal basis with each other and is fundamental to the enjoyment of all other rights (1). It points out that actual or perceived difficulties with decision-making—often associated with persons with mental disabilities—are often used to justify the denial of the exercise of legal capacity and that this is discriminatory because decision-making difficulties can be overcome with support. This support includes supported decision-making to ensure that a person's will and preferences are discerned and given effect. The CRPD Committee states that appropriate supported decision-making will normally be able to assist a person to identify and give effect to their will and preferences. However, it also acknowledges the rare occasions where, despite significant efforts being made, it is not possible to ascertain these then a best interpretation of such will and preferences can be made (1). The Committee's reasoning here is that diagnosis and mental capacity assessments are generally used as thresholds for substitute decisions being made for the individual but that these are often influenced by subjective, and often misconstrued, assessments of what is in that individual's “best interests” which results in discrimination (1). Moreover, the whole purpose of supported decision-making is to assist and enable decision-making capacity rather than diagnosis and capacity thresholds being used to deny such enablement.

It is clear that the Article 12 CRPD objectives are unlikely to be achieved through mental health and capacity laws that give ultimate precedence to clinical or other professional judgment over the will and preferences of persons with mental disabilities. However, this has led to a divergence of often strongly held opinions on how the objectives of Article 12 CRPD can be achieved.

It has been suggested that Article 12 CRPD compliance can be achieved within substitute decision-making arrangements (such as compulsory psychiatric treatment laws and guardianship) provided they are underpinned by human rights-based principles that include a presumption in favor of an individual's will and preferences and clearly delineate the need for, and role of, supported decision-making (2, 3). The CRPD Committee's General Comment No.1, on the other hand, is unequivocal in stating that substitute decision-making regimes and supported decision-making arrangements are incompatible if genuine effect is to be given to the rights, will and preferences of persons with mental disabilities (1). This CRPD Committee stance has received some resistance, largely by clinicians, who have expressed concerns that such an approach has the potential to deny beneficial treatment and put the individual and public at risk (4, 5). However, compelling arguments exist that it is indeed possible for supported decision-making to coercive psychiatric treatment and this will better ensure that basic needs, including the right to the highest attainable standard of health, are properly meet and dignity respected (6, 7).

Arguments also exist that capacity-based mental health laws not only guarantee beneficial treatment where required and safety but also ensure parity of esteem in terms of treatment of persons with physical and mental health conditions (8–11). This viewpoint appears to be relatively popular given that several states in recently reforming their mental health and capacity laws have either retained, or introduced, capacity or capacity-type thresholds for non-consensual interventions (12) [for example, Victoria's (Australia) Mental Health Act 2014 and the Mental Capacity Act (Northern Ireland) 2016].

However, the best approach to navigating the impasse undoubtedly lies in locating supported decision-making within fundamental CRPD message of equal and non-discriminatory enjoyment of rights. In the context of persons with mental disabilities this requires looking beyond human rights simply in terms of limiting unwarranted interventions to the proactive removal of obstacles to full rights enjoyment and the creation of environments that respect and support such enjoyment (13–15). With this in mind this article will therefore now consider the role of supported decision-making in the context of Scotland which is currently undergoing a review of its mental health and capacity laws with consideration of CRPD compliance within its Terms of Reference.^1^

## Support for Decision-Making: Meanings and Objectives

tHE terms “supported decision-making,” “support for decision-making,” and “support for the exercise of legal capacity” are increasingly, and often interchangeably, referred to in literature, policy and practice in connection with pursuing CRPD compliance for persons with mental disabilities. However, from even a cursory view it is not always clear precisely what these terms are supposed to achieve. Used in conjunction with most existing substitute decision-making arrangements in the context of “supported decision-making” and “support for decision-making” might arguably be said to support an individual to participate in but not necessarily to direct decisions made that concern them. Although the CRPD Committee has also referred to “supported decision-making” when interpreting support for the exercise or legal capacity in Article 12 CRPD (1) it is apparent that in CRPD terms the purpose of “supported decision-making” or “support for the exercise of legal capacity” means something else.

### Equal and Non-discriminatory Enjoyment of Human Rights—A New Approach and CRPD Myth Busting

As already stated, the CRPD is clear that persons with disabilities must, in general, be able to enjoy all human rights on an equal basis with others and without discrimination and, where necessary, to be supported to achieve this.^2^ This also applies to the exercise of legal capacity.^3^ In General Comment No. 1 the CRPD Committee explains that support to achieve this equal and non-discriminatory exercise of legal capacity must possess certain essential characteristics. In order to ensure that primacy is given to the person's rights, will and preferences on the same basis as others the support may take many different forms and must be tailored to an individual's decision- making support needs, its substance and uptake must be in the person with disability's discretion and it's availability must not be conditional on mental capacity assessments (1). In essence therefore, supported decision-making as envisaged by the CRPD seeks to place those who face decision-making challenges in the same position as those who do not experience such challenges.

The CRPD reinforcement of the principle of equal and non-discriminatory rights enjoyment goes beyond that identified in previous international human rights instruments. Traditional understandings of this principle have tended to permit differential treatment provided that it can be objectively and reasonably justified (16–19) which invariably has been the case for persons with physical and mental disabilities (1, 20). The CRPD message is essentially that if real equal rights enjoyment is to be achieved then it is necessary to start with a level playing field: persons with physical and mental disabilities who may experience greater practical, institutional and societal challenges with enjoyment of such rights must be supported—whether by, for example, supported decision-making,^4^ reasonable accommodation,^5^ or universal design^6^—to achieve this on an equal basis with other (20). Only when this level playing field has been reached can the restriction of rights, applying the same criteria for all, be considered (1, 20). To start from a position where certain persons are not entitled to full enjoyment of rights because they possess a certain characteristic – in the case of persons with persons with mental disabilities owing to others' perceptions of their capabilities – results in structural inequalities, and thus discrimination, from the outset (1, 20). Somewhat akin to Sen and Nussbaum's capability approach, rights must be viewed not primarily in terms of defining the extent to which they may be limited. They must instead be viewed from the perspective of ensuring that all are able to enjoy a fulfilled life which includes protection from abuse and discrimination (for example, in the context of care and treatment) and appropriate support and services to allow for full and effective inclusion, participation, recovery and rehabilitation (21, 22). This requires a serious reconsideration of existing legal, policy and practice frameworks if there is to be parity of rights enjoyment between persons with mental disabilities and others in respect of enjoyment of all rights and aspects of life (13, 14). However, a full understanding of this approach to equality and non-discrimination is required if states are to successfully navigate the challenges presented by the CRPD Committee's radical assertion that supported decision-making must replace substitute decision-making (15).

Articles 12(3) and 12(4) CRPD collectively identify appropriately tailored supported decision-making as an important safeguard to the equal exercise of legal capacity. Amongst other things, Article 12(4) provides—and this is reinforced by the CRPD Committee's General Comments Nos 1 and 6 (1, 20)—that such safeguards must ensure respect for “the rights, will and preferences of the person” and be “free of conflict of interest and undue influence.” In essence, this means ensuring that an individual's authentic choices are given effect in the same way as others' choices are. At this juncture it is important to remember that enjoyment of rights for anyone may also subject to their limitation provided this is lawful, necessary, proportionate and non-discriminatory.

Strong concerns have been expressed about giving such priority to the will and preferences of persons with mental disabilities. Fears have been expressed that if the CRPD Committee's approach is followed it will allow for no intervention whatsoever and may result in individuals being left without vital support and at risk of harm or likely to cause harm to others (4, 23). However, this is to misunderstand the intention behind the CRPD message. The use of supported decision-making and absence of, as previously mentioned, potentially discriminatory mental capacity assessments leading to substituting the judgment of others in the individual's “best interests” allows for the individual's views to be given effect to the extent only that this would occur with others. Where meaningful communication is genuinely impossible the CRPD Committee recognizes that supported decision-making does encompass the ability for others to make a non-discriminatory best interpretation of the person's will and preferences (1). This interpretation will be something which is very different to a “best interests” decision and would be based on information gathered from those know to the individual and taking into account the person's values and beliefs and past expressions of will and preferences (3, 24, 25). Whilst there is inevitably some debate about whether this in effect amounts to substitute decision-making but by another name (3, 26–28), this does potentially allow for decisions to be made in many challenging situations. Moreover, if a person with mental disability therefore presents a risk of harm to themselves or to others then they would be treated in exactly the same way as persons without such a disability. This might involve steps being taken under civil or criminal law. In crisis situations it might also include taking steps to provide a “breathing” or safe space in which to address the causes of a person's mental distress and to ascertain their genuine will and preferences. This to some extent should ameliorate anxieties around having to give effect to an individual's wishes expressed in times of acute emergency (29) although it is conceded that national civil and criminal justice environments will have to adapt to be conducive to such an approach.

With this template in mind the possibilities supported decision-making and current supported decision-making framework in Scotland will now be considered.

## Supported Decision-Making Possibilities: The Nature and Types of CRPD Supported Decision-Making

Having established that the objective of CRPD supported decision-making is to ensure that an individual's will and preferences are given effect to the same extent as those of others in all situations. Leaving aside for the time being the debate over whether or not this can be achieved within substitute decision-making frameworks, it is worth considering how the CRPD Committee's visualizes supported decision-making.

The CRPD itself^7^ does not state what form such support should take but General Comment No.1^8^ provides a clearer indication of this albeit in broad terms. Important elements of support are specified as encompassing “both informal and formal support arrangements, of varying types and intensity.” and may, for example, include one or more trusted support persons, peer support, advocacy (including self-advocacy support), or assistance with communication, measures relating to universal design and accessibility (involving the state and private individuals or bodies), the provision of information in an understandable format, professional sign language interpretation as well as recognizing diverse, non-conventional methods of communication (especially non-verbal forms of communication) and advance planning.

In contrast to the relatively scant information about the other forms of potential supported decision-making General Comment No. 1^9^ actually says rather more about advance planning. It states that “the ability to plan in advance is an important form of support, whereby [they] can state their will and preferences which should be followed at a time when they may not be in a position to communicate their wishes to others.” It emphasizes the need for equality of opportunity engage in advance planning which might take various forms and that, where desired, support should be provided to assist a person complete the advance planning process. Finally, it directs that the person making an “advance directive,” and no one else, must decide point at which it enters in force (or ceases to have effect). Moreover, it should not come into effect when a person is assessed as lacking mental capacity. This last requirement tends to be at odds with common conceptions of advance statements or directives the operation of which does indeed tend to hinge on mental incapacity assessments.

## Supported Decision-Making in Scotland

### Background

Laws are only one component of giving effect to international human rights standards and resourcing and institutional and societal policies, practices and culture are also essential (15, 30). However, what laws contain and direct are a strong indication of state and societal commitment to human rights adherence (15).

Scotland's laws and practices are currently at a crossroads in terms of support, care and interventions for persons with mental disabilities. It is a devolved region of the United Kingdom (UK) which has incorporated the largely civil and political European Convention on Human Rights (ECHR) rights into its legal framework through the Human Rights Act 1998. Scotland's devolved public bodies and courts must therefore interpret the law and act in accordance with respect for individuals' ECHR rights, and individuals can enforce the rights through national courts and tribunals.^10^ At devolved region level it's legislation and ministerial policy must be ECHR compatible or can be declared unlawful and unenforceable.^11^

At the same time, respecting the UK's international law commitments, any devolved Scottish legislation or policy that places the UK in breach of such obligations may also be prevented by the UK Government.^12^ Adherence to the CRPD obviously falls within these international obligations. Unlike the ECHR, the CRPD is not incorporated into UK law which means that it is influential but its rights are not directly legally enforceable within Scotland. That being said, some indirect CRPD influence can be discerned in terms of the Scottish and wider UK courts not contravening international law unless there is national legislation to the contrary and through any influence the CRPD has on European Court of Human Rights rulings which the UK must follow. Additionally, in Scotland there is already an appetite for at least partial CRPD compliance. Supported decision-making has figured in its current Mental Health Strategy 2017–2027 (31) and in the recent Scottish Government review of the Adults with Incapacity (Scotland) Act 2000. Its influence was central to the recently completed Independent Review of Learning Disability and Autism in the Mental Health Act (32) and, as already mentioned, is a driving force for the current Scottish Mental Health Law Review.

At the time the Scottish Parliament enacted its current mental health and capacity law, the Mental Health (Care and Treatment) (Scotland) Act 2003 (Mental Health Act) and Adults with Incapacity (Scotland) Act 2000 (Adults with Incapacity Act) ECHR compliance was a human rights priority in Scotland. The principles that underpin the implementation of both Acts were therefore strongly ECHR informed. However, the UK subsequently ratified the CRPD which, along with developments in ECHR jurisprudence, added an entirely new dimension to this area of law and practice.

The principles that underpin implementation of both Acts were designed to limit any non-consensual interventions relating to persons with mental disabilities so that they are lawfully authorized, proportionate, the least restrictive alternative and provide a benefit not otherwise attainable.^13^ Moreover, relevant to the issue of respect for an individual's rights, will and preferences, regard must be had for the person's present and past ascertainable wishes and feelings even where a person has been deemed to lack decision-making capacity.^14^ The Mental Health Act also emphasizes the need for patients to participate in care and treatment decisions and to be appropriately supported to do this.^15^ The Adults with Incapacity Act requires those responsible for implementing interventions—including guardians, attorneys and institutional managers—to encourage the exercise and development of the adult's skills relating to their welfare, finances and property.^16^ However, neither Act is particularly clear about how this support should ascertain a person's wishes and feelings or who bearers ultimate responsibility for this (3) and their respective Codes of Practice do not add much to this and mainly refer to only assisting communication (3). Nor does this principle currently carry any greater weight than any of the other principles. That being said, both Acts did take forward recommendations in reports which prompted their enactment that the individual's voice be heard and respected in all decisions made concerning them and that “best interests” assessments, owing to their paternalistic connotations, be omitted from the legislation (33, 34). Moreover, advance planning (in the form of psychiatric advance statements and powers of attorney) and advocacy, both of which are referred to in General Comment No 1, are explicitly mentioned in Scottish mental health and capacity law.

### Advance Planning and Advocacy in Scottish Mental Health and Capacity Legislation

#### Advance Planning: Psychiatric Advance Statements

The Mental Health Act refers to psychiatric advance statements^17^ which allow patients to specify the ways in which they do and do not wish to be treated for their mental disorder should they subsequently lose capacity. The “capacity test” under this legislation is that the patient has significantly impaired decision-making ability because of the mental disorder such decisions pertaining only to care and treatment for the mental disorder.^18^ The Mental Health Tribunal for Scotland and clinicians must have regard to these wishes but can override them provided the Act's aforementioned principles, reinforced by those relating to compulsion,^19^ are adhered to and the reasons for the override are recorded.

International studies indicate that advance statements are often seen as an important component in promoting human rights-based approaches in healthcare (35). There is evidence that psychiatric advance statements improve outcomes in terms of experience of crises, taking medication, reduced coercion, and human rights respect (36–40) even though generally uptake is relatively low (41).

The low uptake of psychiatric statements is mirrored in Scotland (42) despite amendments to the Mental Health Act being introduced in 2015 to encourage the making of advance statements by requiring health boards, overseen by the Mental Welfare Commission for Scotland, to record the existence (and withdrawal) of advance statements and publicize any support they offer to patients to make or withdraw such statements.^20^ They also share many issues that more widely present problems for the CRPD Committee. This is firstly seen in their reliance on mental capacity to make them and incapacity to come into effect and binary nature. This effectively locks patients, once capacity is deemed to have been lost, into decisions made some time ago which may no longer be representative of their current will and preferences. This sits uncomfortably with the Committee's rejection of diagnostic and capacity thresholds although it has alternatively been argued that to deny the ability to enter into such arrangements may actually prevent a patient from expressing their autonomous wishes in crises (43). Secondly, the CRPD seeks to ensure active support for equal rights enjoyment rather than define the perimeters for non-consensual intervention but it is not entirely clear whether this is currently always the motivation behind making advance statements in Scotland. Of course, decisions to override preferences expressed in an advance statement can be challenged by patients on human rights grounds (44). However, as already mentioned, as the current legal framework in Scotland currently gives precedence to ECHR rights and ECHR jurisprudence continues to favor the emphasis on defining limits for intervention approach. For the time being advance statements are therefore likely to be interpreted in accordance with this.

#### Advance Planning: Powers of Attorney

Another recognized form of advance planning can be found in the making and use of financial and welfare powers of attorney which is regulated by the Adults with Incapacity Act.^21^ These allow individuals to both specify the actions they would like to be taken regarding their finances, property, and welfare should they lose capacity—on in the case of financial powers of attorney, if the individual so wishes, whilst they retain capacity—and also to nominate who they would like to exercise these powers on their behalf. Although there are a few areas where it is unclear whether or not the granter can empower the attorney to act on their behalf (45) it is evident from the legislation, and reinforced in its Code of Practice and related professional guidance, that giving expression to the adult's genuine wishes and feelings—which allows for specifying how their incapacity triggering the powers is assessed—is of primary importance (46, 47).^22^

Powers of attorney thus allow for a great deal of autonomy and control for the granter, arguably more so than psychiatric advance statements. Moreover, attorneys are obliged by the Adults with Incapacity to have regard to the Act's principles when exercising their powers which includes respect for the adult's wishes and feelings and supporting and encouraging their skills. However, with the exception of some financial powers of attorney, in CRPD terms they are ultimately subject to the same issues concerning reliance of mental capacity assessments and their binary nature as psychiatric advance statements.

#### Independent Advocacy

Although there is no general right to advocacy across Scotland the Mental Health Act does confer a right to access to independent advocacy to any person who has a mental disorder together with a corresponding duty on local authorities and health boards to ensure this.^23^ This applies whether or not the person is subject to compulsion under the Act. In Scotland, independent advocacy is viewed as a means by which individuals with mental disorder can be supported to have control over their lives through making their own decisions and expressing their wishes (48–50). Both this and the absence of mental capacity assessments activating the right to independent advocacy brings the provision very close to the CRPD requirements for supported decision-making albeit that a diagnosis of mental disorder is still required to access it. Attempts were made in 2015 to reinforce the provision by amending the Mental Health Act to require local authorities and health boards to account to the Mental Welfare Commission for their mental health independent advocacy provision.^24^ The reality, however, is that despite increasing demand for advocacy it is under-resourced with gaps in provision. Providers are therefore forced to strategically allocate their resources mainly to patients subject to psychiatric compulsion (51, 52).

### Mental Welfare Commission Guidance

#### “Soft” Supported Decision-Making

The recognition in legislation of relevant principles and supported decision-making frameworks or arrangements clearly adds weight to, and assists in driving forward, support for the exercise of legal capacity but, of course, such support may also be available outside legislation. For example, following an information gathering exercise, the Mental Welfare Commission for Scotland issued guidance in 2016 on supported decision-making (53) in which it identified several non-legislative (“soft”) forms of supported decision-making currently applicable to Scotland in addition to those that are referred to in the Mental Health Act and Adults with Incapacity Act.

These “soft” forms of supported decision-making include trusted persons (which could include professionals, friends, or families), peer support, advocacy, community and neighborhood support, assistance with, and clear, communication, technological support, and forms of advance planning beyond advance statements and powers of attorney [for example, anticipatory care planning, personal statements and specific decisions about particular types of medical treatment such as DNACPR (Do Not Resuscitate)].

#### Operational Requirements for Supported Decision-Making

The guidance also sets out the operational requirements for effective supported decision-making. This includes the presumption of capacity and functional decision-making assessments to determine point of access to, and type/nature of, supported decision-making, adherence to legislative principles and human rights standards, appropriate time for delivering the support and ensuring no undue influence and conflict of interest. It also highlights the benefits and issues concerning family involvement in terms of ensuring the authenticity of the individual's will and preferences, the fact that the choice of support should be that of individual, clarity as to the provider of the support, honesty, communication and clarity around the options for support and decisions to be made, positive risk taking and record keeping.

#### Guidance Challenges

The aim of the guidance was to give better effect to existing legislative provision and move toward Article 12 CRPD compliance (53). It seeks to achieve this by reminding, instructing and assisting those responsible for the care, treatment and support of persons with mental disabilities of the importance of actively supporting the individual to exercising their legal capacity through ensuring their voice is heard. However, it is evident that the guidance arguably faced the same CRPD compliance challenges that are being more widely experienced in law and practice reform.

Whilst the purpose of the guidance was to try to prevent unnecessary, or at least delay, non-consensual interventions and guarantee the individual's voice the guidance is working within and alongside a substitute decision-making regime. Although the human rights-based legislative principles promote enlightened approaches there are still no absolute guarantees that there will be a presumption in favor of the individual's will and preferences. Moreover, whilst the legislation does not allow for best interests decisions there are indications that these nevertheless do occur in both practice and, sometimes, judicial decisions (54). All the guidance can therefore realistically do is to reinforce the fact that the legislative provisions designed to protect individual autonomy are proactively and expansively given effect.

### Human Rights-Based Approaches: But, Which One?!

The differing approaches of the CRPD and earlier human rights treaties, such as the ECHR, has already been alluded to. This can cause dilemmas given that the incorporation of the ECHR in the UK and Scottish legal frameworks affords its rights greater legal weight than CRPD rights although the CRPD is influential, as mentioned, in Scotland. Indeed, any states which are parties to both the ECHR and CRPD will encounter similar challenges. ECHR jurisprudence has been at pains to emphasize that persons with mental disability have the right to the same level of rights guarantees and safeguards as others. It has, for example, reinforced the functional capacity assessment requirement as opposed to blanket assumptions of mental capacity, the views of a person who has lost mental capacity must not be discounted and proportionality and last resort actions in relation to non-consensual interventions impacting on an individual's liberty and other aspect of their autonomy.^25^ Article 12 CRPD may arguably influence the European Court of Human Rights' interpretation of this, although at present there is little evidence of this occurring to any sizeable degree.^26^ However, the ECHR does seem comfortable with the fact that, despite the safeguards, it might on occasion be necessary and acceptable to justify the restriction of a person's liberty and autonomy on the basis of diagnosis of mental disability or related impairment, including loss of mental capacity.^27^ This obviously potentially prevents that person from enjoying their right to exercise their legal capacity on an equal basis with others which is arguably contrary to the CRPD. That being said, if one adopts the CRPD and CRPD Committee interpretation of equality of rights enjoyment, as outlined earlier, then it might be possible to achieve greater synergy between the two treaties even within the perimeters of mental health and capacity legislation. The focus would be on supporting decision-making, making “best interpretations” where absolutely necessary and only adopting non-consensual interventions where this would be the case for others without a diagnosis of mental disability or mental incapacity assessment. As previously stated, this would lend itself more readily to even difficult cases. However, admittedly more research would be required to ascertain how this might be practically implemented, including the effectiveness and efficacy of different forms of support for decision-making.

## Conclusion: The Way Forward?

It is clear that attempts have been made to support autonomy, through legislative principles and recognition of psychiatric advance statements, powers of attorney and independent advocacy provision, in Scottish legislation. It is also clear that other “soft” supported decision-making mechanisms also exit alongside side which operate in practice. However, the fact remains that respect for the individual's views must currently compete with other principles, practices and societal and institutional cultures that might be more favorable to non-consensual intervention which are linked to diagnosis and mental capacity assessments and, despite legislative direction to the contrary, best interest decisions. The current legal framework in Scotland can probably be most accurately described as adhering to an enlightened medical model. It seeks to maintain and enhance individual autonomy and rights but focuses on protecting those with mental disabilities (and, where necessary, others), but not necessarily their rights, and accepts the potential inevitability of restriction and seeks to define the limits to it. At present, it provides no absolute guarantees in terms of the necessary ingredients to achieve the CRPD interpretation of equality in the exercise of legal capacity. Such ingredients would require support timeously provided at the point of need (which may be required long before considering non-consensual intervention, during and following such intervention) and decisions to restrict a person's autonomy of which are premised on assumptions about capabilities and risk associated with diagnosis or mental capacity. Evidence for elsewhere indicates that even autonomy enhancing arrangements such as the “good man” system in Sweden where only limited possibility exists to substitute another's views for those of a person with mental disability will not meet CRPD requirements (55).

There is an appetite within Scotland to move toward a more CRPD compliant legal framework. The influence of the CRPD in relation to the recent reviews relating to the Adults with Incapacity Act and Learning Disability and Autism in the Mental Health Act have already been mentioned. In addition, in 2016 the Mental Welfare Commission for Scotland and Center for Mental Health and Capacity Law (Edinburgh Napier University) conducted a law reform scoping exercise against an ECHR and CRPD template. In the resultant 2017 report *Scotland's Mental Health and Capacity Law: the Case for Reform* (56) made several recommendations which included that Scottish mental health and capacity law required revisiting, with the active involvement of persons with lived experience (reflecting Article 4(3) CRPD requirements) to ensure it was continuing to meet international human rights standards, notably the ECHR and CRPD. Amongst other things, it noted that the current diagnostic and capacity thresholds in the legislation were problematic in terms of achieving a non-discrimination approach to the care, treatment and support of persons with mental disability. It also recommended that more needed to be done to maximize the autonomy and agency of people with “mental disorder”^28^ as well as consideration of what “respect for rights, will and preferences” actually means. It is perhaps worth noting in this context that other Mental Welfare Commission research has reported that many of the persons with lived experience consulted had indicated that levels of autonomy desired by individuals in relation to care and treatment will vary according to the situation (57).

*The Case for Reform*, together with calls from stakeholders (included lived experience), persuaded the Scottish Government sufficiently that it should establish the previously mentioned Scottish Mental Health Law Review. The Review is seeking to address, amongst other things, the challenging questions posed by the CRPD and General Comment No 1 and the CRPD's interpretation of the right to liberty in Article 14 (58). This includes consideration of what equality and non-discrimination in relation to rights enjoyment looks like in the context of non-consensual psychiatric and other measures. The quality and reliability of mental capacity assessments and efficacy of such assessments as the threshold to non-interventions together with the scope of supported decision-making will also being investigated. Moreover, in terms of achieving CRPD transformation, issues such as actual and perceived levels of risk and the ability of existing ordinary and forensic mental health (and wider criminal justice) systems to adapt to the CRPD “paradigm shift” will need to be explored.

If equal rights enjoyment underpinned by supported decision-making is to be genuinely accessible for persons with mental disabilities in Scotland, and thereby CRPD compliance achieved, then fundamental legal and practice culture change will be required (15). The extent to which it will be possible to achieve this remains to be seen.

## Data Availability Statement

All datasets generated for this study are included in the article/supplementary material.

## Author Contributions

The author confirms being the sole contributor of this work and has approved it for publication.

## Conflict of Interest

The author declares that the research was conducted in the absence of any commercial or financial relationships that could be construed as a potential conflict of interest. The author is a member of the Executive Team of the Scottish Mental Health Law Review. However, the views expressed in this article are those of the author alone and not of the Executive Team. The handling editor declared a past collaboration with the author.

## Footnotes

^1^The Scottish Mental Health Law Review website, including its Terms of Reference, can be found at https://www.mentalhealthlawreview.scot/ The author is a member of the Review's Executive Team. The opinions expressed in this article are not, however, necessarily those of the Review Executive Team or it's Chair, John Scott QC, but of the author alone.

^2^Article 5 CRPD.

^3^Articles 12(1)-(2) CRPD.

^4^Articles 12(3)-(4) CRPD.

^5^Articles 2 and 5 CRPD.

^6^Articles 2 and 4 CRPD.

^7^Articles 12(3) and (4) CRPD.

^8^Paras 17, 18, and 29, General Comment No. 1.

^9^Para 17, General Comment No. 1.

^10^Sections 2, 3, and 6 Human Rights Act 1998.

^11^Sections 29(2)(d) and 57(2) Scotland Act 1998. This differs from England where ECHR incompatible laws can only be formally declared incompatible but remain enforceable until amendment or repeal by the UK Parliament (section 4 Human Rights Act 1998).

^12^Sections 35(1)(a) and 58(1) Scotland Act 1998.

^13^Sections 1(3)(e) - (h) and 1(4), 36(5), 44(4), and 64(5) Mental Health Act; Sections 1(2)-(3) Adults with Incapacity Act.

^14^Section 1(3)(a) Mental Health Act; Section 1(4)(a) Adults with Incapacity Act.

^15^Sections 1(3)(c)-(d) Mental Health Act.

^16^Section 1(5) Adults with Incapacity Act.

^17^Sections 275-276C Mental Health Act. Unlike in England, advance statements relating to physical health are not statutorily recognized in Scotland although it is considered that they would be accepted as valid, and thus effect given to them, under the common law (30).

^18^Sections 36(4)(b), 44(4)(b), and 64(5)(d) Mental Health Act.

^19^Sections 36(5), 44(4), and 64(5) Mental Health Act.

^20^Sections 276A−276C Mental Health Act (inserted by section 21 Mental Health (Scotland) Act 2015).

^21^Sections 15–24 Adults with Incapacity Act.

^22^Sections 15(3)(c)(iii) and 16(3)(c)(iii) Adults with Incapacity Act.

^23^Section 259 Mental Health Act.

^24^Section 259A Mental Health Act [inserted by section 27 Mental Health (Scotland) Act 2015].

^25^*Winterwerp v Netherlands* (1979-80) 2 EHRR 387; *Shtukaturov v Russia* (App no 44009/05) (2012) 54 EHRR 27; *Sykora v Czech Republic* (App no 23419/07) (2012) ECHR 1960; *Stanev v Bulgaria* (2012) 55 EHRR 22; *DD v Lithuania* (2012) ECHR 254; *A-MV v Finland* (App no 53251/13) ECtHR, 23 March 2017.

^26^See, for example, *A-MV v Finland (ibid)*.

^27^*Winterwerp v Netherlands* (*op cit*).

^28^The term “mental disorder” is used in both the Mental Health Act and Adults with Incapacity Act. Section 328 of the Mental Health Act defines “mental disorder” as meaning mental illness, personality disorder.
